# 1372. Impact of Age, Gender, Ethnicity, Comorbidities, and Vaccination Status on Long COVID in a Brooklyn Community

**DOI:** 10.1093/ofid/ofad500.1209

**Published:** 2023-11-27

**Authors:** Calvin Ta

**Affiliations:** Maimonides Medical Center, Brooklyn, New York

## Abstract

**Background:**

A significant number of patients have been affected by COVID-19 and experience prolonged symptoms. Several studies have documented the long-term effects of COVID-19, however little is known about the symptom severity, expected clinical course, or how long it takes to return to baseline health. At our institution, we have continued to care for patients at Maimonides Medical Center Adult Post-COVID Clinic (MMCPCC) who have persistent post-COVID symptoms.

**Methods:**

We conducted a retrospective chart review of patients at MMCPCC from November 2020 to June 2022. Patients 18 years or older, with confirmed or suspected COVID-19, and whose symptoms persisted beyond four weeks were included. We evaluated the demographics, comorbidities, vaccination status, reported post-COVID symptoms, the duration of these symptoms, and history of COVID-19 hospitalization. The average number of symptoms (ANOS) and hospitalization rate (HR) were measured.

**Results:**

Twenty-five long COVID symptoms were reported and evaluated. The most common symptoms were malaise/fatigue, dyspnea, anxiety, arthralgia/myalgia, and headache. Of 173 patients that were included in our study, 49.7% (86) were white, 60.1% (104) were female, 42.2% (73) were 50-69 years old, and 80.9% (140) were unvaccinated at the time of COVID-19 infection. Of the vaccinated patients, 18.6% were white, 23.3% were Black, 23.5% were Hispanic, and 11.8% were Asian. The ANOS was 4.37 and the HR was 24.9%. 50 to 59-year-olds reported the highest ANOS (5.33). Asian patients reported the lowest ANOS (3.29). Minority patients and patients with obesity had higher HRs (35.1% and 35.2% respectively). Hispanic and Black patients with ≥ 2 comorbidities (41.2% and 25.6% respectively) had an ANOS of 5.57 and 4.55 respectively. 60.7% (105) of patients reported symptoms > 3 months and 24.3% (42) reported symptoms for over a year. Of those, 72.1% of Black and 70.6% of Hispanic patients had long COVID for > 3 months compared to 59.3% of white and 35.3% of Asian individuals.

Number of comorbidities among the racial groups with long COVID at MMCPCC.

**Figure:**
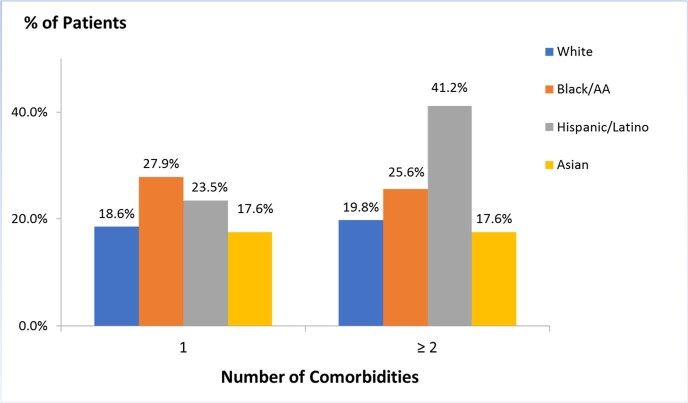

Patients with long COVID greater than 3 and 12 months among the racial groups at MMCPCC.

**Figure:**
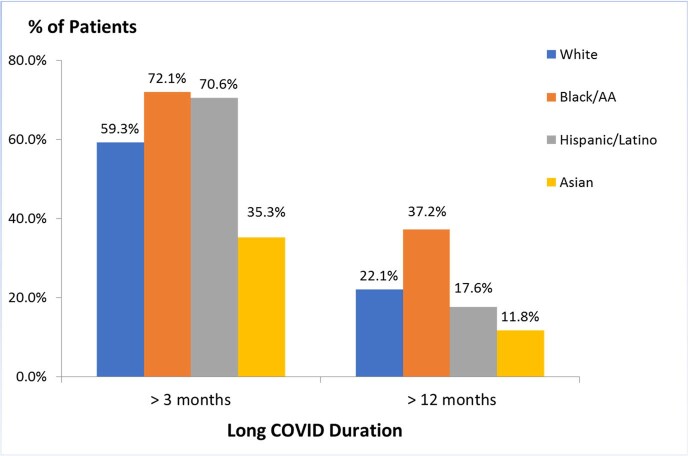

List the most common long COVID symptoms that were evaluated.

**Figure:**
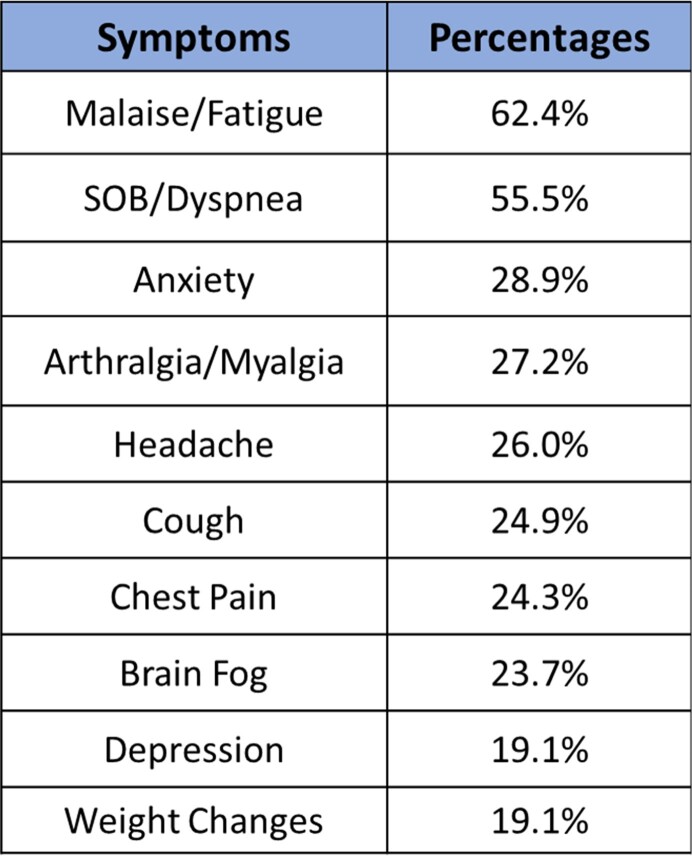

**Conclusion:**

Our study showed unvaccinated, 50 to 69 years old, white, and female patients were predominantly affected by long COVID. Hispanic and Black patients with multiple comorbidities have higher ANOS, 71.7% had long COVID for > 3 months, and 31.7% had long COVID over a year.

**Disclosures:**

**All Authors**: No reported disclosures

